# Investigation of the Inter‐ and Intrascanner Reproducibility and Repeatability of Radiomics Features in T1‐Weighted Brain MRI

**DOI:** 10.1002/jmri.28191

**Published:** 2022-04-09

**Authors:** Rosalind Nina Mitchell‐Hay, Trevor S. Ahearn, Alison D. Murray, Gordon D. Waiter

**Affiliations:** ^1^ Department of Radiology, Aberdeen Royal Infirmary NHS Grampian Aberdeen UK; ^2^ Aberdeen Biomedical Imaging Centre University of Aberdeen Aberdeen UK

**Keywords:** radiomics, repeatability, reproducibility, neuro‐imaging

## Abstract

**Background:**

Radiomics is the high throughput analysis of medical images using computer algorithms, which specifically assess textural features. It has increasingly been proposed as a tool for the development of imaging biomarkers. However, an important acknowledged limitation of radiomics is the lack of reproducibility of features produced.

**Purpose:**

To assess reproducibility and repeatability of radiomics variables in brain MRI through a multivisit, multicenter study.

**Study Type:**

Retrospective.

**Population:**

Fourteen individuals visiting three institutions twice, 10 males with the mean age of 36.3 years and age range 25–51.

**Field Strength:**

3D T1W inversion recovery on three 1.5‐T General Electric scanners.

**Assessment:**

Radiomics analysis by a consultant radiologist performed on the T1W images of the whole brain on all visits. All possible radiomics features were generated.

**Statistical Test:**

Concordance correlation coefficient (CCC) and dynamic range (DR) for all variables were calculated to assess the test–retest repeatability. Intraclass correlation coefficients (ICCs) were calculated to investigate the reproducibility of features across centers.

**Results:**

Of 1596 features generated, 57 from center 1, 15 from center 2, and 22 from center 3 had a CCC > 0.9 and DR > 0.9. Eight variables had CCC > 0.9 and DR > 0.9 in all centers. Forty‐one variables had an ICC of >0.9. No variables had CCC > 0.9, DR > 0.9, and ICC > 0.9.

**Data Conclusion:**

Repeatability and reproducibility of variables is a significant limitation of radiomics analysis in 3DT1W brain MRI. Careful selection of radiomic features is required.

**Level of Evidence:**

4

**Technical Efficacy Stage:**

2

Radiomics is the high throughput analysis of medical images using computer algorithms, producing quantifiable texture features.^1^ It attempts to quantify features of an image that humans use when assessing an image through generating the features using novel mathematical constructs. These features have been shown to correlate with underlying pathological and genetic findings.^1^, ^2^ In glioblastoma, an 11‐feature radiomics model derived from MRI data was shown to outperform established predictive models.^3^ MRI‐based predictive models have also been developed in advanced nasopharyngeal cancer and have been shown to improve prognostic ability.^4^ A radiomics approach has similarly been shown to correlate with clinical endpoints in multiple other pathologies including gynecological and colorectal cancer.^5^ Over the past few years, there has been an exponential rise in the number of publications which involve radiomics with the aim of developing clinically relevant imaging biomarkers, highlighting the scientific interest in the topic.^6^

Important limitations of radiomics include its variable and unknown reproducibility and repeatability in different applications.^7^, ^8^ In this paper, reproducibility refers to the ability to replicate radiomic features in a different center while repeatability refers to the ability to replicate measurements when taken under the same conditions in the same center.

There are multiple areas where variation can be introduced within a typical radiomics workflow, including the initial image acquisition, the pre and postprocessing of images, the segmentation, and the calculation of the radiomic features themselves. The Image Biomarker Standardization Initiative (IBSI) is an international collaboration which was established in part to address these issues.^9^ The collaboration recently published validated, consensus‐based reference values for 169 features^9^ based upon an analysis of T1‐weighted MRI images, computed tomography (CT) and flurodeoxyglucose–positron emission tomography (PET) images of 51 patients. However, there is limited guidance on how to account for any variation introduced into the radiomic workflow through differences in hardware, software, and sequence parameters employed to acquire the images used for analysis.

The acknowledged limitation of repeatability is likely to be more pronounced within MRI when compared to other imaging modalities due to inherent difficulties in its standardization. The values assigned to voxels in MRI do not reflect defined substances as is the case in other modalities such as in the Hounsfield scale reflecting the atomic number in CT. When compared to the number of published radiomic analyses, there is a relative paucity of studies investigating these limitations in MRI. In a test–retest and image registration single center study of MRI in patients with glioblastoma, it was reported that there were multiple confounders within the image acquisition, including image registration and field bias.^8^ Another study, using a publicly available prostate MRI dataset, has shown that radiomic features vary in their reproducibility and that there are multiple factors affecting this.^10^ While these studies have highlighted issues in reproducibility of radiomics, demonstrating the confounders within image acquisition, they do not specifically discuss scanner variability. A clearer understanding of this will guide further research and aid in future clinical applications of radiomics.

Thus, the aim of this study is to analyze the intra and interscanner variability of radiomics features extracted from a previously created high‐resolution T1W dataset.

## Methods

### Image Acquisition

All participants gave signed informed consent and the study received appropriate ethics approval, for the initial study, which included future analysis. All participants were native English speakers, right‐handed (self‐reported), met the standard MRI safety criteria and had no history of diagnosed neurological disorder, major psychiatric disorder or treatment with psychotropic medication, including treatment for substance misuse. The participants were not paid, but they were reimbursed for expenses. All participants provided written informed consent and the study was approved by the local research ethics committee. The Calibrain study was initially carried out to assess the inter and intrascanner repeatability of functional MRI and to facilitate future standardized protocol development for multicenter studies.^11^, ^12^ Within this study, 14 individuals visited three centers twice within a time period of 2 weeks. All participants each underwent two brain MRIs on 1.5‐T General Electric (GE) scanners with all parameters remaining consistent across all sites, minor differences are acknowledged and these exist due to variations within both the scanner software and hardware. At center 1, a GE 1.5‐T Signa Nvi/CVi scanner (software version 9.1) was used; at center 2, a GE Signa LX scanner (software version 9.1 M4), and at center 3, a GE 1.5‐T Signa scanner (software version 11M3/11M4SP1) was used. A standard quadrature head coil was used at each site.^11^, ^12^, ^13^

From the sequences acquired, the high‐resolution T1W images were acquired using 3D inversion recovery prepared fast gradient echo volume sequences. The parameters used are shown in Table 1.^13^ The Oxford Centre for Functional Magnetic Resonance Imaging of the Brain Software Library (FSL) brain extraction tool was used to remove all nonbrain tissue from the acquired images and to create individual masks for the entirety of the remaining brain tissue.^14^, ^15^ The automatic segmentation tool also removed the eye and optic nerve.

**TABLE 1:** Acquisition Parameters Performed According to the Different Centers

Centre	Orientation	Repetition Time (msec)	Echo Time (msec)	Slice Thickness With no Gap (mm)	Inversion Time (msec)	Field of View (mm^2^)	Matrix	Flip Angle (°)	Number of Slices
1	Coronal	5.9	1.9	1.7	600	200	256 × 256	15	128
2	Coronal	8.2	3.3	1.7	600	200	256 × 256	15	128
3	Coronal	5.9	1.4	1.7	600	200	256 × 256	15	128

### Radiomics Analysis

Radiomics analysis was performed using the open‐source software Pyradiomics^16^ using Spyder v3.3.6^17^ within Anaconda v1.18.^18^ The analysis was performed by a consultant radiologist with 8 years of clinical experience. All features which were generated fall within the list defined in the IBSI reference manual.^9^ Pyradiomics allows for customization of the output dependent on the imaging modality used. All studies were processed in the same way. The code used was those suggested within pyradiomics^9^ for evaluating MRI with 3‐mm slices.^16^ The histogram of intensity values was normalized to a scale of 100 with a fixed bin width of 5 applied.

Within the radiomics analysis, a number of image filters were applied to the original images with radiomic features then calculated from these postprocessed images as well as from the original. The filters that were applied were wavelet [this returns eight images covering all combinations of low (L) and high (H) pass filtering across the imaged spectrum], Laplacian of Gaussian (Log) with filter widths 2, 3, 4, and 5, square, square root, logarithm, and exponential, respectively.

In this study, the following features classes were calculated for each filtered image: first‐order, gray level dependencies matrix, gray level co‐occurrence matrix (GLCM), gray level size zone matrix (GLSZM), gray level run‐length matrix (GLRLM), and the neighboring gray tone difference matrix (NGTDM).

### Statistical Analysis

R studio version 4.0.0^19^ was used for statistical analysis with tidyverse, ggpubr, rstatix, car, broom, readxl, ggplot2, purrr, lpsolve, ggpubr, knitr, QuantPsyc, irr, janitor, pysch, DescTools, and DynRB packages used.

The concordance correlation coefficient (CCC) was initially calculated for individual centers to quantify the repeatability between the two scans performed on each subject.^20^ In those found to have a CCC of >0.9, the normalized dynamic range (DR) was then calculated. The DR quantifies the intersubject variability: with a range from 0 to 1. Features with a higher DR are more desirable as differences are more discernable while still being repeatable. Within this study, following work by Segel et al and Balagurunathan et al, the thresholds of a DR > 0.9 and CCC > 0.9 were applied to determine the variables with good repeatability.^21^, ^22^

Intraclass correlation coefficients (ICCs) were calculated based upon the subjects first visit, to investigate the reproducibility of features across centers. The ICC was calculated within the psych module of the statistical package and a two‐way random effects model was used. In accordance with Koo et al, the reproducibility was defined as excellent if ICC was >0.9; good if ICC was >0.75 and <0.9; moderate if ICC was >0.5 and <0.75; and poor if ICC was <0.5.^23^ The ICC results were then assessed according to the feature class and the image filter applied in preprocessing.

## Results

Of the 1595 radiomics features calculated, 22 from center 1, 57 from center 2, and 12 from center 3 had a CCC > 0.9 and DR > 0.9. Eight features were common in data analyzed from all centers. None of the radiomic features calculated with the original, wavelet‐LHH, wavelet‐HHL, wavelet‐HHH, square root, or exponential image filters were found to have good test–retest repeatability. The log‐sigma image filters resulted in a higher number of features (*n* = 8) with CCC > 0.9 and DR > 0.9, with image filter log‐sigma‐5 mm the highest with three features. When divided according to feature class, no features within the NGTDM class had a CCC > 0.9 and a DR > 0.9. The GLCM feature class had the highest number of features with seven features with both CCC > 0.9 and DR > 0.9 in the three centers individually and when combined. The breakdown according to image type and feature class is detailed in Table 2.

**TABLE 2:** Breakdown of Features Obtained With a CCC > 0.9 and DR > 0.9 Split According to Image Filter Applied and Feature Class of the Radiomic Variable

	Number of Features With CCC > 0.9 and DR > 0.9 (Percentage %)			
	Center 1	Center 2	Center 3	Combined
Image filter (*n*)				
Original (107)	0 (0.00)	0 (0.00)	0 (0.00)	0 (0.00)
Wavelet‐LLH (93)	0 (0.00)	2 (3.51)	1 (6.25)	0 (0.00)
Wavelet‐LHL (93)	1 (4.55)	1 (1.75)	0 (0.00)	0 (0.00)
Wavelet‐HLL (93)	1 (4.55)	4 (7.02)	0 (0.00)	0 (0.00)
Wavelet‐HLH (93)	0 (0.00)	1 (1.75)	1 (6.25)	0 (0.00)
Wavelet‐LHH (93)	0 (0.00)	0 (0.00)	0 (0.00)	0 (0.00)
Wavelet‐LLL (93)	4 (18.18)	4 (7.02)	0 (0.00)	0 (0.00)
Wavelet‐HHL (93)	0 (0.00)	0 (0.00)	0 (0.00)	0 (0.00)
Wavelet‐HHH (93)	0 (0.00)	0 (0.00)	0 (0.00)	0 (0.00)
Log‐sigma‐2 mm (93)	3 (13.64)	7 (12.28)	2 (12.50)	1 (12.50)
Log‐sigma‐3 mm (93)	3 (13.64)	12 (21.05)	4 (25.00)	2 (25.00)
Log‐sigma‐4 mm (93)	3 (13.64)	12 (21.05)	3 (18.75)	2 (25.00)
Log‐sigma‐5 mm (93)	7 (31.82)	13 (22.81)	4 (25.00)	3 (37.50)
Logarithm (93)	0 (0.00)	1 (1.75)	0 (0.00)	0 (0.00)
Square (93)	0 (0.00)	0 (0.00)	1 (6.25)	0 (0.00)
Square root (93)	0 (0.00)	0 (0.00)	0 (0.00)	0 (0.00)
Exponential (93)	0 (0.00)	0 (0.00)	0 (0.00)	0 (0.00)
Feature class				
Shape (14)	0 (0.00)	0 (0.00)	0 (0.00)	0 (0.00)
First order (18)	5 (22.73)	11 (19.30)	3 (18.75)	1 (12.50)
GLCM (24)	8 (36.36)	20 (35.09)	11 (68.75)	7 (87.50)
GLDM (14)	0 (0.00)	4 (7.02)	2 (12.50)	0 (0.00)
GLRLM (16)	7 (31.82)	17 (29.82)	0 (0.00)	0 (0.00)
GLSZM (16)	2 (9.09)	5 (8.77)	0 (0.00)	0 (0.00)
NGTDM (5)	0 (0.00)	0 (0.00)	0 (0.00)	0 (0.00)

The final three letters L or H denote low or high, respectively.

CCC = concordance correlation coefficient; DR = dynamic range; GLDM = gray level dependencies matrix; GLCM = gray level co‐occurrence matrix; GLSZM = gray level size zone matrix; GLRLM = gray level run‐length matrix; Log = Laplacian of Gaussian; NGTDM = neighboring gray tone difference matrix.

The eight radiomic features which had a CCC > 0.9 and DR > 0.9 across all centers are detailed in Table 3. GLCM correlation appears four times with different filters, GLCM information measure of correlation appears three times while the first‐order feature root mean squared appears once.

**TABLE 3:** List of Radiomics Features With CCC > 0.9 and DR > 0.9 Across All Three Centers and Their Respective CCC and DR Values

Radiomic Feature	Center 1		Center 2		Center 3	
	CCC Value	DR Value	CCC Value	DR Value	CCC Value	DR Value
Log‐sigma‐2‐0‐mm‐3D_glcm_Correlation	0.97	0.95	0.93	0.95	0.94	0.95
Log‐sigma‐3‐mm‐3D_glcm_Correlation	0.950	0.97	0.98	0.96	0.98	0.96
Log‐sigma‐3‐mm‐3D_glcm_Imc2	0.94	0.98	0.94	0.98	0.94	0.98
Log‐sigma‐4‐0‐mm‐3D_glcm_Correlation	0.94	0.98	0.97	0.98	0.99	0.98
Log‐sigma‐4‐0‐mm‐3D_glcm_Imc2	0.93	0.99	0.92	0.99	0.93	0.99
Log‐sigma‐5‐0‐mm‐3D_firstorder_RootMeanSquared	0.93	0.98	0.97	0.99	0.91	0.99
Log‐sigma‐5‐0‐mm‐3D_glcm_Correlation	0.93	0.99	0.94	0.99	0.95	0.99
Log‐sigma‐5‐0‐mm‐3D_glcm_Imc2	0.91	0.99	0.93	0.99	0.91	0.99

Radiomic features are listed in the form: Imagetype_Class_Featurename.

CCC = concordance correlation coefficient; DR = dynamic range; GLCM = gray level co‐occurrence matrix; Log = Laplacian of Gaussian; Imc2 = information of correlation measure 2.

**FIGURE 1:**
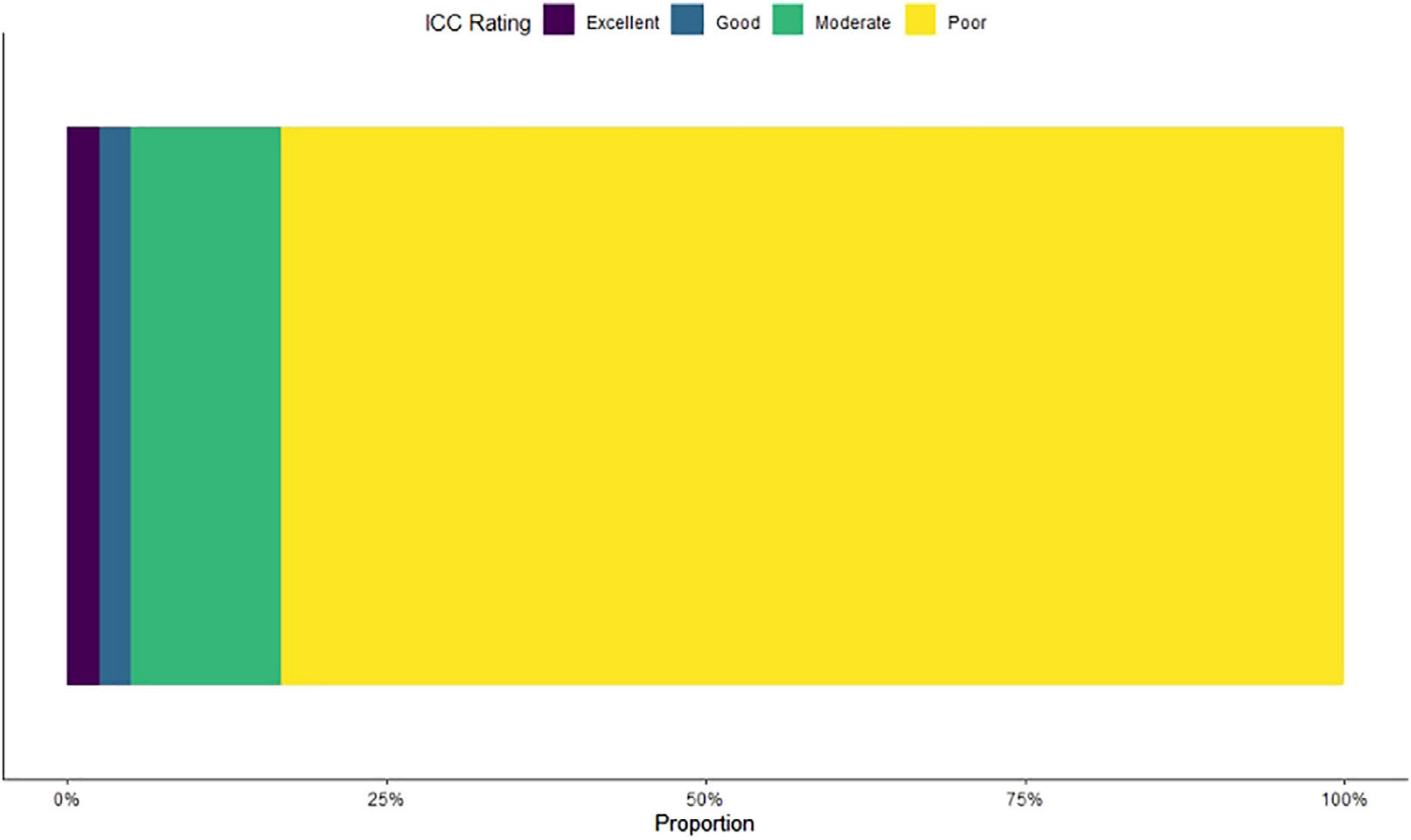
Reproducibility of radiomic features across the centers using interclass correlation coefficient (ICC)

Within the cross‐center ICC calculation from the 1595 radiomics features calculated, 3% were rated excellent (*n* = 40), 2% were rated good (*n* = 39), 12% were rated moderate (*n* = 188), and 83% were rated poor (*n* = 1328). These results are summarized in Fig. 1, while Table 4 lists all the features found to have an excellent correlation across all three centers. There was no overlap between the features that had a CCC and DR > 0.9 and those that had an excellent ICC.

**TABLE 4:** List of Radiomic Features With an Excellent Reproducibility Across the Different Centers and Their Respective ICC Values

Radiomic Feature	ICC Value
Original_shape_Maximum2DDiameterRow	0.95
Original_shape_Maximum3DDiameter	0.97
Original_shape_MeshVolume	0.93
Original_shape_VoxelVolume	0.93
Original_glrlm_RunLengthNonUniformity	0.94
Wavelet‐LLH_firstorder_Energy	0.94
Wavelet‐LLH_firstorder_TotalEnergy	0.93
Wavelet‐LLH_glrlm_RunLengthNonUniformity	0.94
Wavelet‐LHL_firstorder_Energy	0.93
Wavelet‐LHL_firstorder_TotalEnergy	0.93
Wavelet‐LHL_ngtdm_Coarseness	0.94
Wavelet‐LHH_firstorder_Energy	0.93
Wavelet‐LHH_firstorder_TotalEnergy	0.93
Wavelet‐HLL_firstorder_Energy	0.93
Wavelet‐HLL_firstorder_TotalEnergy	0.93
Wavelet‐HLL_glrlm_RunLengthNonUniformity	0.95
Wavelet‐HLL_ngtdm_Coarseness	0.93
Wavelet‐HLH_firstorder_Energy	0.93
Wavelet‐HLH_firstorder_TotalEnergy	0.93
Wavelet‐HLH_glrlm_RunLengthNonUniformity	0.90
Wavelet‐HLH_ngtdm_Coarseness	0.93
Wavelet‐HHL_firstorder_Energy	0.93
Wavelet‐HHL_firstorder_TotalEnergy	0.93
Wavelet‐HHL_ngtdm_Coarseness	0.91
Wavelet‐HHH_firstorder_Energy	0.93
Wavelet‐HHH_firstorder_TotalEnergy	0.93
Wavelet‐LLL_glrlm_RunLengthNonUniformity	0.97
Log‐sigma‐2‐0‐mm‐3D_firstorder_Energy	0.93
Log‐sigma‐2‐0‐mm‐3D_firstorder_TotalEnergy	0.93
Log‐sigma‐2‐0‐mm‐3D_glrlm_RunLengthNonUniformity	0.91
Log‐sigma‐2‐0‐mm‐3D_ngtdm_Coarseness	0.96
Log‐sigma‐3‐mm‐3D_firstorder_Energy	0.93
Log‐sigma‐3‐mm‐3D_firstorder_TotalEnergy	0.93
Log‐sigma‐4‐0‐mm‐3D_firstorder_Energy	0.93
Log‐sigma‐4‐0‐mm‐3D_firstorder_TotalEnergy	0.93
Log‐sigma‐5‐0‐mm‐3D_firstorder_Energy	0.92
Log‐sigma‐5‐0‐mm‐3D_firstorder_TotalEnergy	0.92
Squareroot_glrlm_RunLengthNonUniformity	0.92
Exponential_firstorder_Energy	0.94
Exponential_firstorder_TotalEnergy	0.94

Features are listed in the form: Imagetype_Class_Featurename. The final three letters L or H denote low or high, respectively.

ICC = interclass correlation coefficient; Log = Laplacian of Gaussian; GLCM = gray level co‐occurrence matrix; GLRLM = gray level run‐length matrix; NGTDM = neighboring gray tone difference matrix.

**FIGURE 2:**
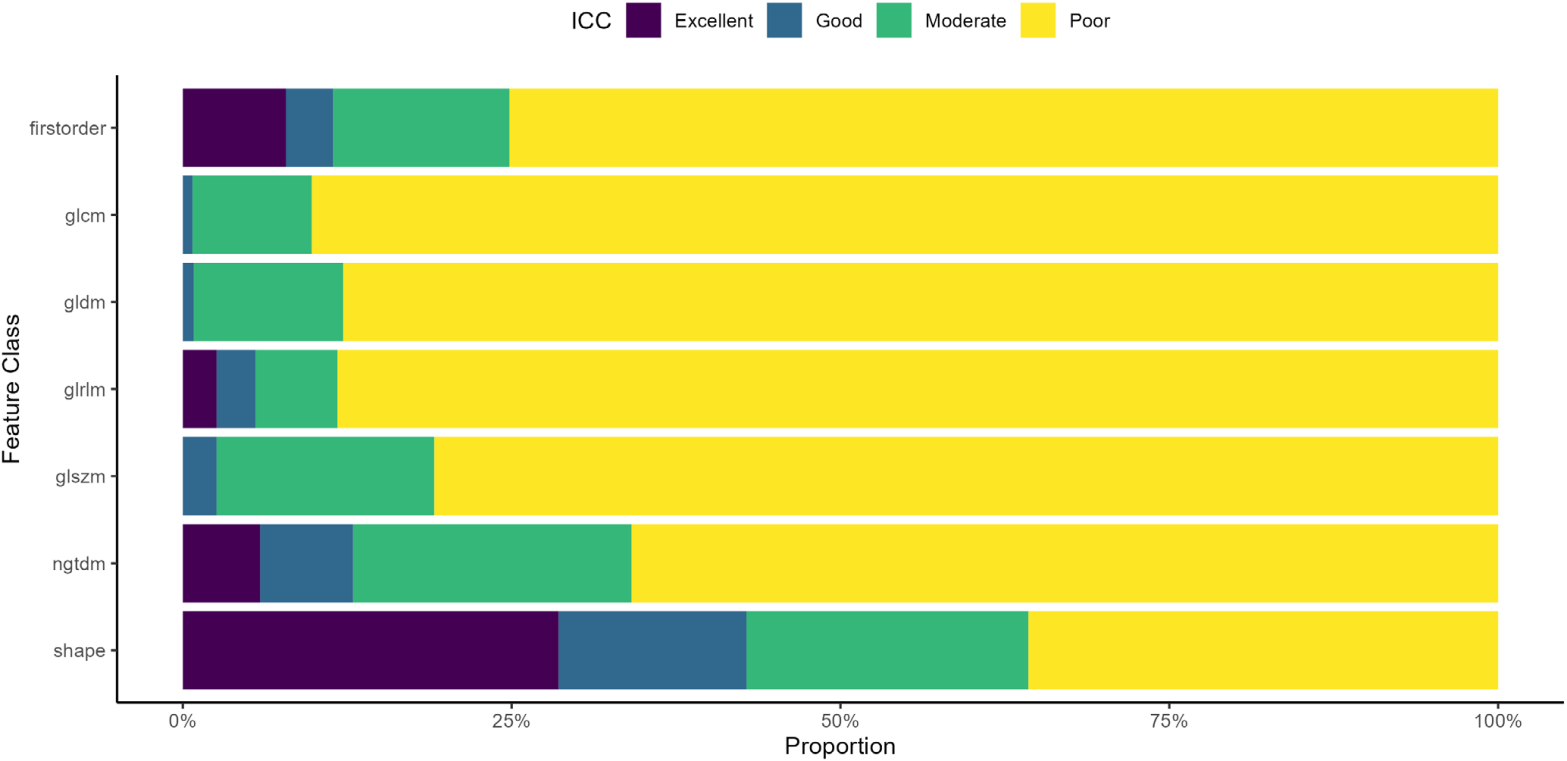
Reproducibility of radiomic features as split by feature class using interclass correlation coefficient (ICC)

When the ICC values were then separately analyzed by feature class, the shape and first‐order features had the highest proportion of reproducible features classed as excellent. In contrast, the GLCM had the highest proportion of poor features. This is summarized in Fig. 2.

**FIGURE 3:**
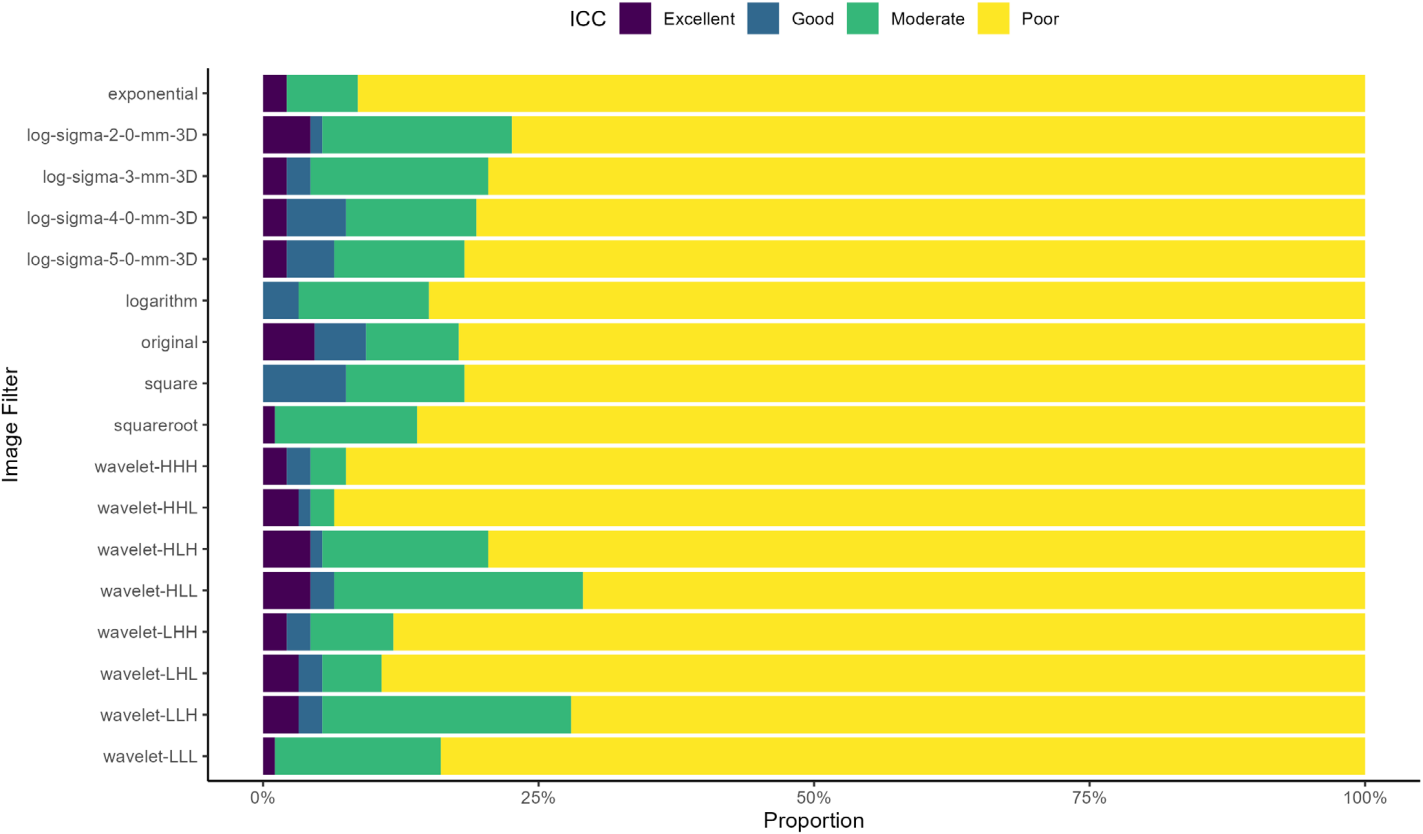
Reproducibility of radiomic features divided by image filter applied using interclass correlation coefficient (ICC)

When divided according to the image filter applied in preprocessing, there was a relatively even spread throughout the different image filters, as summarized in Fig. 3. The original image filter had both the highest proportion (5%) of features with an excellent ICC as well as the highest proportion with a poor ICC (88%).

## Discussion

In our study, >80% of the features calculated had a poor reproducibility (ICC < 0.5) while only 3% had excellent reproducibility. This demonstrates the variation that exists within radiomic features acquired on 1.5‐T scanners from the same manufacturer. It reinforces the findings from a ground truth simulation phantom study that highlighted the need for care when combining studies to create large datasets when those studies have been performed using different imaging parameters and scanners.^24^ With the increasing use of large datasets for radiomics analysis, heterogeneity within image acquisition and variety of scanners used is to be expected. It is important that this is addressed to produce clinically reproducible parameters and that such variation is accounted for within future analysis. This is particularly pertinent within prognostic modeling in radiomics to prevent the inclusion of features which are not true prognostic indicators.

Within the test–retest data in this study, <5% of features showed excellent repeatability (CCC > 0.9 and DR > 0.9) in all three centers. When comparing features with a CCC > 0.9 and DR > 0.9, only 8 were repeatable across all three centers. Of those eight features, two occurred more than once with different image filters (correlation, lmc2) while root squared mean appeared once. Both correlation and lmc2 fall within the GLCM feature class which, with first‐order features, were shown to have the highest percentage of features with both CCC and DR >0.9 across all centers. This is consistent with a test–retest study in T2W images in rectal cancer that found features from the GLCM class and first‐order features had low measurement error when compared to the higher order feature classes.^25^ Previous studies have acknowledged and investigated variation within GLCM features providing recommendations for the standardization of GLCM features: such as use of one quantization method,^26^ one GLCM size,^26^ and use of invariant feature,^27^ There is however limited work examining the repeatability and reproducibility in MRI of all the large number of features produced by radiomics analysis. This study aimed to investigate this and, in doing so, highlight the need for further research. Efforts were made to adhere to previous recommendations where feasible, invariant features however were not calculated as they were not standard within the open‐source software. One potential source for the large variation identified maybe inherent field inhomogeneity. While further research should first be carried out to reduce variation through other methods such as intensity normalization it is a possibility that this limits the application of all radiomics features in MRI.

In a previous test–retest study in giloblastoma multiforma using T2 fluid attenuated inversion recovery and T1W imaging data, it was found that the wavelet and Log image filters had features with the highest ICC.^8^ This study concluded that radiomic features may be helpful in differentiating between progression and pseudo progression, provided that the variables are carefully selected with appreciation of those which are most reproducible.^8^ This finding is similar to our results obtained in T1W brain images, which also demonstrate that Log image filters have the greatest reproducibility. This is different to a previous study looking at radiomics applied to apparent diffusion coefficient (ADC) and T2W images in prostate MRI that showed that wavelet features had low repeatability.^10^ Within cervical cancer, the features derived from images with filters applied were not found to be substantially different to unfiltered images.^28^ These disparate results highlight the need for increased understanding of the variation which exists within radiomics. Care should be taken to select not just the most reproducible radiomics features but also whether to apply image filters.

In regard to the repeatability of different feature classes: shape and first‐order features were found have the highest proportion of repeatable features. This finding is not surprising as the use of these features within image analysis is established and well‐documented with measurements of tumor size often included in formalized oncological staging criteria.^29^ When comparing these results across the three centers, the GLRLM feature, run‐length nonuniformity, and the NGDTM feature, coarseness appears multiple times across different image filters with the remaining features belonging to either the shape or first‐order feature class. The relatively increased repeatability of coarseness when compared to other higher order features has also been noted in a study by Gourtisoyianni et al.^25^ Coarseness is an indication of the spatial rate of change in intensity of the area of interest.^9^, ^30^ From its mathematical formula, it is inversely related to the first‐order characteristic uniformity which may explain its relatively higher repeatability when compared to the other features from its class. Run‐length nonuniformity reflects the distributions of runs (adjacent voxels with same gray level score) over the length of a matrix as defined by the area of interest within the segmented image.^9^, ^31^ The lower the value the more equally distributed the run lengths are. Related features, such as the normalized run‐length nonuniformity, did not show high values of repeatability in this study.

While the relative stability of features is required for imaging biomarkers, it is not the only important characteristic: the ability for the feature to discriminate is also important and is not addressed here. Of note, and pertinent to this, the run length nonuniformity which within our study was shown to have excellent ICC across scanners was found to have poor discrimination when measured on ADC images from sarcoma and oropharyngeal carcinoma.^32^ In a study looking at radiomic feature robustness in nasopharyngeal carcinoma within PET CT, it was shown that features could be highly clinically useful but also have a poor ICC.^33^ The importance of this point was further highlighted by Kim et al who demonstrated, in the context of low grade gliomas, that when a lower threshold for concordance was applied, the resultant model had improved diagnostic accuracy.^34^ However, when considering the roadmap for the development of imaging biomarkers, it is precision, including repeatability and reproducibility, that needs to be proved.^35^ Within the context of the roadmap, a poor ICC suggests a poor precision and the active exclusion of features with poor ICC should be considered. A new selection‐based approach has recently been published which integrates the assessment of feature stability with the radiomics feature selection rather than as separate process.^36^ When this approach was compared to a separated methodology in three different multicenter imaging cohorts, this integrated approach was shown to yield improved performance.^36^ This study highlights the need for consideration of scanner variability within the developing methodology in radiomics research.

### Limitations

This study is limited by the small number of subjects and single sequence; however, every effort was made to standardize the imaging parameters and the multicenter nature makes the results a useful addition to the research addressing the reproducibility of radiomic features. However, this does mean that comparison can only be made on broad terms with different sequences. In addition, this study did not include analysis of tissues containing any pathology, instead of normal tissue. Most disease processes are known to demonstrate heterogeneity and complexities that would not be present within normal issues. Given the bias of radiomics toward nonhomogeneous intensities, it would not be unreasonable to argue that performance may improve when performed on pathological tissues.

## Conclusion

The repeatability and reproducibility of radiomics features is an important limitation of radiomics analysis in MRI.
